# Frederick Hoffman's Essay on the Dissimilarity of Fixed Vegetable Alkaline Salts, with Observations and Remarks on the Changes Which the Septic Acid Undergoes by Combination with Those Alkalies

**Published:** 1804-05-01

**Authors:** 


### Frederick Hoffman's Essay *on the Dissimilarity of* Fixed Vegetable Alkaline Salts, *with Observations and Remarks on the Changes which the Septic Acid undergoes by Combination with those Alkalies, by Dr.* Mitchill.

It has been repeatedly objected to me, that the *nitrous* acid of the shops does not correspond with the character which I find to be possessed by the *septic* acid. It is true that, in some of my early essays, published during the years 1795 and onward to 1798, I did employ the term "nitrous" as a sort of synonime to the word "septic:" but then I always enclosed it within a parenthesis, and my object was to lead the mind towards that acid as having a radical similitude with the one I was describing, though without any intention of affirming their identity. I now find that this has been misconstrued, and that some persons understand me as having affirmed that the nitrous and septic acids were so exactly alike, that what was true of one was in all respects true of the other.

Now, this is a serious mistake in me, if ever I said so, or in others, if they have ascribed to me what I did not say. In 1796 (see Med. Rep. vol. ii. p. 235—240) I stated this difference, as I thought, very plainly, and at considerable length. I repeated this sentiment, with some variation of statement, in 1797 (ibid. vol. i. p. 217.) To these I added a number of other considerations in 1799 (ibid. vol. iii. p. 14 & seq.), showing the dissimilitude of these two acid products. More was offered on this point in 1801 (ibid. vol. iv. p. 184-5), exhibiting still further the difference between the acid formed among corrupting materials, and the acid obtained by decomposition of salt-petre. In the same volume, p. 190 and 262, there are other views of this question, intended to put my meaning out of doubt.

That part of the history of putrefaction which I have attempted to explore, and to which I have called the attention of the learned world, *is the series of phenomena which occur from the moment of the formation of septic acid by any of the processes which produce it, to the fixation or neutralization of it by pot-ash.* My attention was directed to this period, because it had been overlooked by almost every observer, as it yet is by the greater part of scientific men; although it certainly embraces the most important portion of the facts connected with animal and vegetable disorganization. The history of the *nitrous acid had, I well knew*

*from*

*from the beginning, been laboriously and profoundly explored.* With that I professed to have little to do, but employed myself chiefly in investigating the character and operation of *the acid of septon,* before it underwent the alteration which gives it the qualities possessed by the *nitrous.*

Still it is taken for granted by the philosophers of our day, that the acid obtained by decomposition and distillation of salt-petre is the very same and identical thing which was formed in the midst of corruption, and among putrefying materials, by a chemical combination of septon and oxygen. This is as confidently asserted by them as if it was a matter of fact. Indeed, it is so absolutely credited and taken for granted, that the *natural history* of the acid, from the time of its formation to the time of its association with pot-ash (which is the most interesting by a great difference), has been almost wholly overlooked; while its *artificial history,* after its separation from pot-ash (by far less important), has attracted an uncommon share of attention.

But men of science did not always confound things at this rate. There was a time when opinions were stated as such, and not obtruded upon the world roundly and positively for realities.

Fourscore years ago the German chemists knew that salt-petre, formed from *distilled nitrous* acid and pot-ash, by synthesis, in a laboratory, differed from the neutral salt produced when septic acid combined with that alkali by the natural process, in heaps of dung and rubbish. Ludwig had remarked, that notwithstanding all attempts to saturate them exactly, there would be some acrimony left in this *nitrum regeneratum;* to correct which a portion of quick-lime must be added.

The difference between natural salt-petre and regenerated salt-petre was so considerable, that the latter was by some affirmed to be unfit for making gun-powder. It was declared to have too little strength for that purpose. Others, however, were of a different sentiment, and declared *regenerated* nitre as good as the *natural.* (Junckeri Conspectus Chemiæ, tab. lxi.)

Stahl knew the destructibility of pot-ash: for when salt-petre was exposed to a torturing fire, for a long time, in an open vessel, it was wholly dissipated; both the acid and alkali disappearing. And he also knew, that if a mixture of salt-petre and charcoal was deflagrated in a tubulated retort, that both the acid and the alkali would be destroyed or decompounded; scarcely a vestige of pot-ash being found in the retort, or of nitrous acid in the receiver.

Kunkel too has ascertained, by experiment, that putrefying blood afforded both septic acid and fixed alkali enough to form salt-petre. From one hundred pounds of corrupted blood, he obtained, by evaporation and crystallization, more than five pounds of genuine nitre. Juncker himself was perfectly aware of the convertibility of alkaline salt to nitre, by exposure to fœtid exhalations.

When an alkaline salt has been exposed too long to intense fire, the watery parts are expelled, and the salt itself degenerates to an earthy, fixed, and insipid consistence.

The following curious fact is told of the convertibility of nitrous acid into pot-ash:—If the alkali, in its dryest state, is mixed with spirit of nitre, and turned to *nitrum regeneratum*, and afterwards, by aid of charcoal in close vessels, this factitious salt-petre is decomposed and turned back to *nitrum fixum*, or pot-ash, there will be found remaining a much larger quantity than was originally made use of. They both contain septon, and their constitution is probably more nearly allied in many particulars than is usually supposed.

It is the vulgar opinion that acids and alkalies are the opposites of nature, because of the effervescence they make on uniting, &c. but the Stahlians were of a different belief, thinking that their readiness to combine with each other indicated rather a similarity of constitution and a homogeneity of corpuscules. (Juncker. Consp. tab. vii.)

There is something uncommonly singular in the limestone caverns which furnish the salt-petre earths in Virginia, North-Carolina, and Tennesse. These excavations are composed altogether of rocky strata of calcareous earth, or carbonat of lime. They extend for considerable distances towards the centre of the mountain in whose sides they exist. Neither rain nor sun-shine ever reaches them. In the bottom of them a salt-petre earth is found, which, after lixiviation, if carried back and replaced, becomes gradually impregnated with salt-petre again. There are no corrupting materials here to supply septic acid. There is no process of incineration to afford pot-ash. All that is discoverable is the crumbling and mouldering of the calcareous rocks which form the caverns. And in this rubbish or scrapings of the floors are found a large portion of *calcareous*, and a smaller quantity of *alkaline* nitre. To detach the portion of acid which is associated with the lime, the hunters and salt-petre makers find it necessary to add a parcel parcel of wood-ashes to the earth brought out of the caves. The alkali of the ashes attracting the acid from its calcareous basis, furnishes a greater quantity of salt-petre, and of a better quality than can be procured by any other method. This *artificial* nitre, added to that previously formed by the *natural* process, makes up the amount which is procured. From a careful consideration of the facts afforded by the *natural* process of salt-petre forming in these vast subterranean excavations, there appears no other way of accounting for the production of both a *portion of the native pot-ash and all the nitrous acid, than by the decomposition of the calcareous rocks,* and the formation of the two materials of salt-petre from the constituent parts of these strata of lime-stone; a part of which seems to change into pot-ash, and another part into nitrous acid!

A somewhat similar fact occurs with regard to the conversion of volatile alkali into nitrous acid. If corruption, instead of going on in open air, is made to take place in a closed apparatus, the ammoniac gradually disappears, and the mass changes to a sulphureo-terrene, or, in some measure, *to a nitrous consistence.* This is analogous to the changing of nitrous acid to ammoniac during the solution of tin, and in other experiments.

Beecher plainly affirms the mixture of septic matter with the water of the ocean. He supposes a quantity of nitrous matter derived to it from the various kingdoms of Nature, whereby salt-petre is superadded to its common salt. This corresponds to Newman's experiment of obtaining *aqua regia* from sea-salt, and of Pringle's experiments of some of it promoting corruption. (See Trans. of American Philosophical Society, vol. v. p. 140.)

But above all the pieces that I have met with in the older writers, *Hoffman's Essay on the dissimilar Nature and Properties of Fixed Alkaline Salts* contains the most pointed evidence of difference between pot-ash after it *has been* combined with septic acid, and pot-ash which has *never been* so combined. His observations are so remarkable that I deem them worthy of a full translation:—" It has been hitherto firmly believed, by experienced chemists, that the nature of the lixivial salts prepared by *calcination* and *incineration,* was not different, but the same, and that they possesed *equal* fitness for the purposes of medicine and the arts; and, indeed, this would seem to be the case, if we merely consider their mixture with acids of various strength, their effervescence, their saturation, and conversion into *salia salsa,* or neutral salts. But it will be more clear-

 ly ly and undeniably evident, from experiments I am about to relate, that the constitution, composition, and qualities of these alkalies is *not the same,* and that a peculiar and specific difference prevails between them.

"1. *Nitrum fixum,* or pot-ash prepared from salt-petre, deflagrated with charcoal, undergoes strong ebullition on having oil of vitriol poured upon it, and exhales a disgusting smell like aqua fortis; but this does not happen if the experiment be made with salt of tartar or of wood-ashes.

"2. If salt of tartar, or alkaline salt (as pot-ash is called by way of distinction), is melted in a crucible, and powdered charcoal be added to it in its melted state, to the amount of about half the quantity of the pot-ash, then the mass, on being poured out, is of a reddish colour, of a fœtid, sulphureous smell, not unlike the *hepar sulphuris* ordinarily prepared from salt of tartar and common brimstone: but if the *fixed nitre,* or that caustic salt which is prepared with regulus of antimony and salt-petre, is melted, and charcoal dust added to it in fusion, it does not undergo the smallest alteration of colour, smell or taste, but remains white and very pure.

"3. If with the salt of tartar, which is usually prepared by an extemporaneous process of burning together two parts of salt-petre and one of tartar, there be mixed a tolerably strong spirit of vitriol, instantly an odour like aqua fortis rises; but this never happens with pure salt of tartar, or any lixivial salt prepared from vegetables by incineration.

"4. There is a remarkable difference between the salt of tartar, prepared from crude tartar, *with* an admixture of salt-petre, and that obtained *without* it, as well as between the former and lixivial salt itself; in this respect particularly, that if you pour oil of vitriol gently and gradually upon the former, a stinking, disagreeable odour arises, and the surface becomes covered with a blackish pellicle, together with rising froth; and, indeed, the body of the liquor itself becomes blackish; but most certainly these appearances never happen to pot-ash.

"5. Clear glass can never be made from salt of tartar and sand; but glass of elegant transparency can be formed from the alkaline product of salt-petre and tartar (§ 3), and likewise from pot-ash.

"6. There exists no doubt among those who are addicted to chemical researches, that a highly caustic and alkaline salt may be prepared from two parts of salt-petre and one of regulus of antimony, melted together by a strong strong fire. This, freed from foreign particles by solution in water, and afterwards dried, acquires an *intensely red* colour by being mixed and digested with highly rectified spirit of wine. But if the like spirit of wine be poured upon the caustic alkaline salt prepared from two parts of salt-petre and one of tartar, dissolved and purified by water in the same manner, there is no tinge or colour of red to be seen.

"And although a tincture of extraordinary virtue, and of a *golden* colour, may be obtained by pouring highly rectified spirit of wine upon the salt of tartar prepared from *tartar alone*; yet the like never happens when the experiment is made with pot-ash, or with that salt of tartar which is formed from *salt-petre* and tartar.

"7. The waters of many of the medicated springs which are cold, and called *acidulous*, or are hot, and called *thermæ*, on undergoing a gentle evaporation, deposit a lixivial salt, which nearly resembles other salts: in this, however, there is a difference, that these lixivial salts of the springs, on being melted with powdered charcoal, are changed to *hepar sulphuris*: but this has not been observed to happen when the like experiment was made with the caustic alkali by regulus of antimony, nitrum fixum, or the alkali from salt-petre and tartar.

"These experiments amply and satisfactorily prove that *common salt of tartar is widely different* from the *alkaline salt which is an ingredient of salt-petre*: for the former, by affusion of oil of vitriol, salutes the nostrils with a fœtid smell, forms a less transparent glass, partly mingles with spirit of wine, and strikes a colour which the nitrum fixum, or the alkali from salt-petre and tartar never do; while, on the other hand, those alkalies which are ingredients of salt-petre, exhale, on being mingled with oil of vitriol, *the nasty odour of aqua fortis*.

"Hence it is correctly inferred, that salts, and other bodies of a very fixed nature, may conceal within them volatile, sulphureous and oily particles, by an attraction too strong for the *most intense fire to overcome*; while, at the same time, their connection is such, that, by virtue of the *attractions* consequent upon mixture with other bodies, these hidden materials may regain their distinct characters, and burst again upon the view."

As these facts were published as long ago as 1736, it might be presumed that the gentlemen who undertake to judge of scientific discussions were acquainted with them. But finding of late, that even they who criticise and publish lish opinions to the world knew nothing about them, I determined to exhibit them in an English version. Why are all these instructive phenomena forgotten and overlooked? Wherefore are the truths disregarded or rejected which our predecessors laboured so hard to accumulate?

But to show the distinction in this case the stronger, the authority of Boerhaave might be cited. This great chemist affirmed (2 El. Chem. proc. 134) in the account he gives of Glauber's spirit of nitre, "that there was never any acid discovered, in any single experiment, like the acid procured by that process." And I am of the same opinion. This acid, and all its derivatives, are certainly *artificial* products, and exist no where but in the laboratories and in well-stopped vessels; while the original septic acid exists copiously among the ruins of organic matter all over the earth.

On the whole, I wish the gentlemen who attend to this subject to consider two things; first, that pot-ash, or the vegetable alkali, assumes an endless variety of forms, without being adulterated by foreign ingredients, and is not that simple, uncompounded element which some of them pretend to think it: the other is, that not a particle of *nitrous* acid ever existed in Nature; in the laboratory of nature *septic* acid is formed, and oftentimes associated to pot-ash; and by that association, and by the process employed to extract it *by art*, THE SEPTIC ACID IS CHANGED TO THE NITROUS. And it is a most happy event for mankind that it is so; for thereby an alteration is wrought both in the constitution of the acid and of the alkali, very beneficial to living beings. Such being the facts, it clearly follows, that all the experiments made upon *nitrous acid and its derivatives* have no bearing or application to the *original septic acid as engendered or evolved in the putrefactive process*. The man must be very dull who affirms that *nitrous compounds* are the common agents of pestilential mischief; though he must be still more dull if he denies *oxygenated septon*, in its various forms, to be the noxious cause which alkaline salts and earths universally neutralize and subdue.

Through want of attention to these points of difference, it is probably to be understood wherefore my meaning has been so often perverted. Without intending to think or to reason wrong, the philosophical men have charged me with mistakes from which I hope I am free.

I request that they who have misunderstood me may peruse this piece, and reconsider what they have written and and read. Particularly I request this of Christopher Albert, who printed a Dissertation at Erlangen in 1798, *de Luis Bovillæ Origine et Naturâ;* and of Nathaniel Weeks, who defended an Essay *de Flava Febre*, at Edinburgh, 1799. I would make a similar request of George Lee, of Philadelphia, if death had not removed him from us; though this amiable and candid man declared to me, in a letter written a little before he became disabled by his infirmities, *that he would never contradict me again*. I give the same invitation to Humphry Davy, of the Royal Institution, and to the Monthly Reviewers, of London; to D. J. C. Smyth, to Mr. Guyton, and to all the advocates for destroying contagion by fumigation with nitrous vapour and oxygenated muriatic gas, on both sides of the Atlantic; and to the writers of speculations upon infectious diseases, who have honoured either myself or my doctrine with their notice, in Philadelphia or Boston. As to the critical remarks made upon Dr. Bay's Dissertation on Dysentery, in Med. Rep. vol. i. p. 241, they were inserted at my suggestion and request, by the learned and polite gentleman who penned that Review. When chemists shall have been taught, by further experiments, how ignorant they are of the true constitution of *nitrous acid*, and, from additional observations, how much they have yet to learn concerning the exact composition of the *vegetable fixed alkali*, they will lay aside a part of the positiveness with which they have conducted this discussion, and practise somewhat more of condescension and forbearance.

*New York, March 20, 1803.*

